# Influence of hemoadsorption during cardiopulmonary bypass on blood vesicle count and function

**DOI:** 10.1186/s12967-020-02369-x

**Published:** 2020-05-15

**Authors:** Lukas Wisgrill, Christian Lamm, Lena Hell, Johannes Thaler, Angelika Berger, Rene Weiss, Viktoria Weber, Harald Rinoesl, Michael J. Hiesmayr, Andreas Spittler, Martin H. Bernardi

**Affiliations:** 1 Comprehensive Center for Pediatrics, Department of Paediatrics and Adolescent Medicine, Division of Neonatology, Paediatric Intensive Care & Neuropaediatrics, Medical University of Vienna, Vienna, Austria; 2 Department of Surgery, Research Labs, Medical University of Vienna, Vienna, Austria; 3 Clinical Division of Haematology and Haemostaseology, Department of Medicine I, Medical University of Vienna, Vienna, Austria; 4 Christian Doppler Laboratory for Innovative Therapy Approaches in Sepsis, Department for Biomedical Research, Danube University Krems, Krems, Austria; 5 Department of Anesthesia and Intensive Care Medicine, Landeskrankenhaus Feldkirch, Feldkirch, Austria; 6 Division of Cardiac Thoracic Vascular Anaesthesia and Intensive Care Medicine, Medical University of Vienna, Waehringer Guertel 18-20, 1090 Vienna, Austria; 7 Core Facility Flow Cytometry, Medical University of Vienna, Vienna, Austria

**Keywords:** Blood vesicle, Cardiopulmonary bypass, Extracellular vesicles, Hemoadsorption, Microvesicles

## Abstract

**Background:**

Extracorporeal circulation during major cardiac surgery triggers a systemic inflammatory response affecting the clinical course and outcome. Recently, extracellular vesicle (EV) research has shed light onto a novel cellular communication network during inflammation. Hemoadsorption (HA) systems have shown divergent results in modulating the systemic inflammatory response during cardiopulmonary bypass (CPB) surgery. To date, the effect of HA on circulating microvesicles (MVs) in patients undergoing CPB surgery is unknown.

**Methods:**

Count and function of MVs, as part of the extracellular vesicle fraction, were assessed in a subcohort of a single-center, blinded, controlled study investigating the effect of the CytoSorb device during CPB. A total of 18 patients undergoing elective CPB surgery with (n = 9) and without (n = 9) HA device were included in the study. MV phenotyping and counting was conducted via flow cytometry and procoagulatory potential was measured by tissue factor-dependent MV assays.

**Results:**

Both study groups exhibited comparable counts and post-operative kinetics in MV subsets. Tissue factor-dependent procoagulatory potential was not detectable in plasma at any timepoint. Post-operative course and laboratory parameters showed no correlation with MV counts in patients undergoing CPB surgery.

**Conclusion:**

Additional artificial surfaces to the CPB-circuit introduced by the use of the HA device showed no effect on circulating MV count and function in these patients. Larger studies are needed to assess and clarify the effect of HA on circulating vesicle counts and function.

*Trial registration* ClinicalTrials.Gov Identifier: NCT01879176; registration date: June 17, 2013; https://clinicaltrials.gov/ct2/show/NCT01879176

## Background

Cardiopulmonary bypass (CPB) surgery appears to influence the host immune response leading to a systemic inflammatory response with increased pro- and anti-inflammatory cytokines [1, 2]. Thus, systemic inflammation might negatively influence the postoperative course of this high-risk patient group [3].

The novel hemoadsorption (HA) device CytoSorb (CytoSorbents Europe GmbH, Berlin, Germany) was designed to adsorb mid-molecular weight hydrophobic molecules, including cytokines, through size exclusion and nonspecific surface adsorption in peripheral blood [4, 5]. The CytoSorb adsorber consists of porous polymerized divinylbenzene beads, encased in a polycarbonate cartridge with a total volume of 300 mL. Though some case reports and case studies suggest promising effects, including effective adsorption of mid-molecular weight hydrophobic molecules in septic shock, there is still a lack of clinical studies and understanding of the device [6–9]. In our previous randomized-controlled trial, the HA device had no impact on the proinflammatory cytokine response and, furthermore, on the postoperative course in patients undergoing cardiac surgery on CPB [4].

Microvesicles (MV), as part of the extracellular vesicle (EV) population are submicron vesicles (0.1–1 µm) secreted or shed from the cellular membrane during activation, proliferation and apoptosis. Upon circulation of whole blood over adsorbent polymers, blood cell activation, adhesion, and release of MVs can occur [10, 11]. Subsequent immune activation and induced apoptosis may lead to the release of apoptotic bodies (AB), defined as being larger than 1 µm [12]. Circulating vesicles thus are receiving increased interest as inflammatory and coagulation biomarkers in clinical settings and might shed light onto new pathophysiological mechanisms [13].

Thus, we hypothesized that the systemic immune activation during CPB could induce MV release and, consequently, that the Cytosorb device—with the proposed “adsorbing” properties—may diminish the release of those immunomodulatory vesicles. Therefore, the aim of this study was to determine the influence of the CytoSorb HA device on the release of peripheral blood MVs in patients undergoing major cardiac surgery on CPB compared to a control group for the first five postoperative days. High-sensitive flow cytometry was used to analyze MV and AB subsets in platelet-free plasma. Additionally, a MV-tissue factor (TF) activity assay was used to investigate TF-bearing vesicles. Polymer-dependent cell activation and adhesion was determined by scanning electron microscopy.

## Methods

### Ethical statement

This study was a subcohort analysis from the randomized control trial as recently published by Bernardi et al. [4]. The study was approved by the ethics committee of the Medical University of Vienna with reference number EK Nr: 1095/2013, reported to the Austrian Federal Office for Safety in Health Care (INS-621000-0505) and registered at ClinicalTrials.Gov (NCT01879176) before recruitment started. Written informed consent was obtained from each patient prior to inclusion. Since analysis of MV characterization was not planned initially, the collection of additional blood samples was amended to the study protocol and approved by the ethics committee on June 16, 2014.

### Study design and patient characteristics

The original study [4] was conducted during September 10, 2013, and May 6, 2015, at the Division of Cardiac Thoracic Vascular Anaesthesia and Intensive Care Medicine, Medical University of Vienna, Vienna, Austria. Additional collection of blood samples for MV characterization started on September 29, 2014. For a detailed description of the trial as well as detailed clinical follow-up we refer to the clinical trial publication [4].

Briefly, patients undergoing elective open-heart surgery (coronary artery bypass graft, valve surgery or combined procedure) with an expected CPB duration of more than 120 min were included. We excluded a priori patients who declined informed consent, underwent transplant surgery, pulmonary endarterectomy or scheduled insertion of a cardiac assist device as well as emergency or urgent procedures. Also, patients with elevated preoperative serum creatinine > 177 µmol/L, C-reactive protein > 20 mg/L, or bilirubin > 34.2 µmol/L; patients with a body mass index < 18 kg/m^2^, pregnancy, history of stroke, receiving chemotherapy, antileukocyte drugs, tumor necrosis factor-α blockers, immunosuppressive drugs (e.g. tocilizumab), or diagnosed with any disease state that could produce leukopenia (e.g. acquired immune deficiency syndrome) were excluded.

In total, 46 adult patients were recruited into the clinical trial. Since analysis of MV characterization was not planned initially, we only included 18 patients (n = 9 CytoSorb group, n = 9 Control group) starting on September 29th 2014. Patient characteristics were collected by case report form and data are summarized in Table 1.

**Table 1:** Patient and surgical characteristics

	Control n = 9	CytoSorb n = 9	*p*-value
Patient characteristics			
Female	2 (22.2%)	2 (22.2%)	1.000
Age (years)	72.0 [66.0; 75.0]	71.0 [70.0; 78.0]	0.791
Height (cm)	174 [162; 180]	179 [176; 180]	0.658
Body weight (kg)	83.0 [60.0; 96.0]	91.0 [78.0; 96.0]	0.268
BMI (kg/m^2^)	26.2 [23.4; 28.9]	28.7 [26.2; 32.1]	0.216
Preoperative risk indicators			
Myocardial infarction	0 (0%)	0 (0%)	1.000
Asthma	0 (0%)	0 (0%)	1.000
COPD	0 (0%)	1 (11.1%)	1.000
NIDDM	2 (22.2%)	4 (44.4%)	0.620
IDDM	2 (22.2%)	0 (0.00%)	0.471
CKD	0 (0%)	0 (0%)	1.000
Cardial decompensation	0 (0%)	0 (0%)	1.000
PAOD	1 (11.1%)	1 (11.1%)	1.000
aHTN	5 (55.6%)	6 (66.7%)	1.000
Angina pectoris (no)	7 (77.8%)	7 (77.8%)	1.000
Angina pectoris (stable)	1 (11.1%)	2 (22.2%)	
Angina pectoris (instable)	1 (11.1%)	0 (0.00%)	
LVEF > 50%	7 (77.8%)	7 (77.8%)	1.000
LVEF 30–50%	1 (11.1%)	2 (22.2%)	
LVEF > 30%	1 (11.1%)	0 (0.00%)	
Surgical characteristics			
CABG	3 (33.3%)	2 (22.2%)	0.689
Valve procedure	5 (55.6%)	4 (44.4%)	
Combined procedure	1 (11.1%)	3 (33.3%)	
Anesthesia duration (min)	425 [367; 446]	431 [363; 550]	0.453
Procedure duration (min)	326 [281; 352]	324 [265; 420]	0.825
CPB duration (min)	142 [134; 181]	149 [141; 203]	0.691
Aortic cross-clamp (min)	115 [74.0; 129]	122 [95.0; 149]	0.627
Fibrinogen (g)	0.00 [0.00; 2.00]	2.00 [0.00; 2.00]	0.291
Thrombocytes (units)	0.00 [0.00; 0.00]	0.00 [0.00; 0.00]	1.000
PCC (LU)	0.0 [0.0; 1.0]	0.0 [0.0; 0.5]	0.874
FFP (units)	0 (0%)	0 (0%)	1.000
Packed red blood cells (units)	0.00 [0.00; 1.00]	0.00 [0.00; 0.00]	0.695
Crystalloids (L)	4.8 [4.0; 5.0]	4.5 [4.3; 5.4]	0.689
Colloids (L)	0.5 [0.5; 0.6]	0.5 [0.5; 0.5]	0.884

Data are presented as median [IQR] or as frequency (n (%))

*BMI* body mass index, *CABG* coronary artery bypass, *COPD* chronic obstructive pulmonary disease, *NIDDM* non insulin dependent diabetes mellitus, *IDDM* insulin dependent diabetes mellitus, *CKD* chronic kidney disease, *PAOD* peripheral artery occlusive disease, *PCC* prothrombin complex concentrate, *aHTN* arterial hypertension, *AP* angina pectoris, *LVEF* left ventricular ejection fraction, *FFP* fresh frozen plasma

### Study procedure and blood sampling

Anesthesia and CPB priming were performed according to institutional standards. CPB was performed by using non-pulsatile flow at 2.5 l/min/m^2^, a non-heparin-coated circuit, and a membrane oxygenator (Quadrox; Maquet, Hirrlingen, Germany, or Capiox; Terumo, Eschborn, Germany). More procedural details can be found in the previously published study protocol [4].

In the intervention group, the 300 mL CytoSorb cartridge was installed into the CPB circuit using a side arm coming from the venous outflow tube and returned over the venous reservoir prior to the oxygenator. The cartridge flow was standardized controlled by a roller pump with 200 mL/min. The control group was treated similar without installed adsorber. Blood samples were taken at following timepoints: preoperative; before CPB; after CPB; 2 h after CPB; 24 h after CPB; 48 h after CPB and on the 5th post-operative day (POD). Blood sampling was conducted as described in our published protocol [14]. Briefly, whole blood was carefully drawn into sodium citrated blood collection tubes and immediately carefully transported into the laboratory. For the subcohort analysis, additional blood samples for MV characterization were drawn according to published standards and once centrifuged at 2500×*g* for 15 min at room temperature without brake [14]. The resulting platelet-poor plasma was gently transferred into a polypropylene tube and centrifuged at 13,000×*g* for 5 min at room temperature. The platelet-free plasma was distributed in aliquots, shock frozen in liquid nitrogen and subsequently stored at − 80 °C prior to analysis.

### Immunolabeling and flow cytometry

**Fig. 1:**
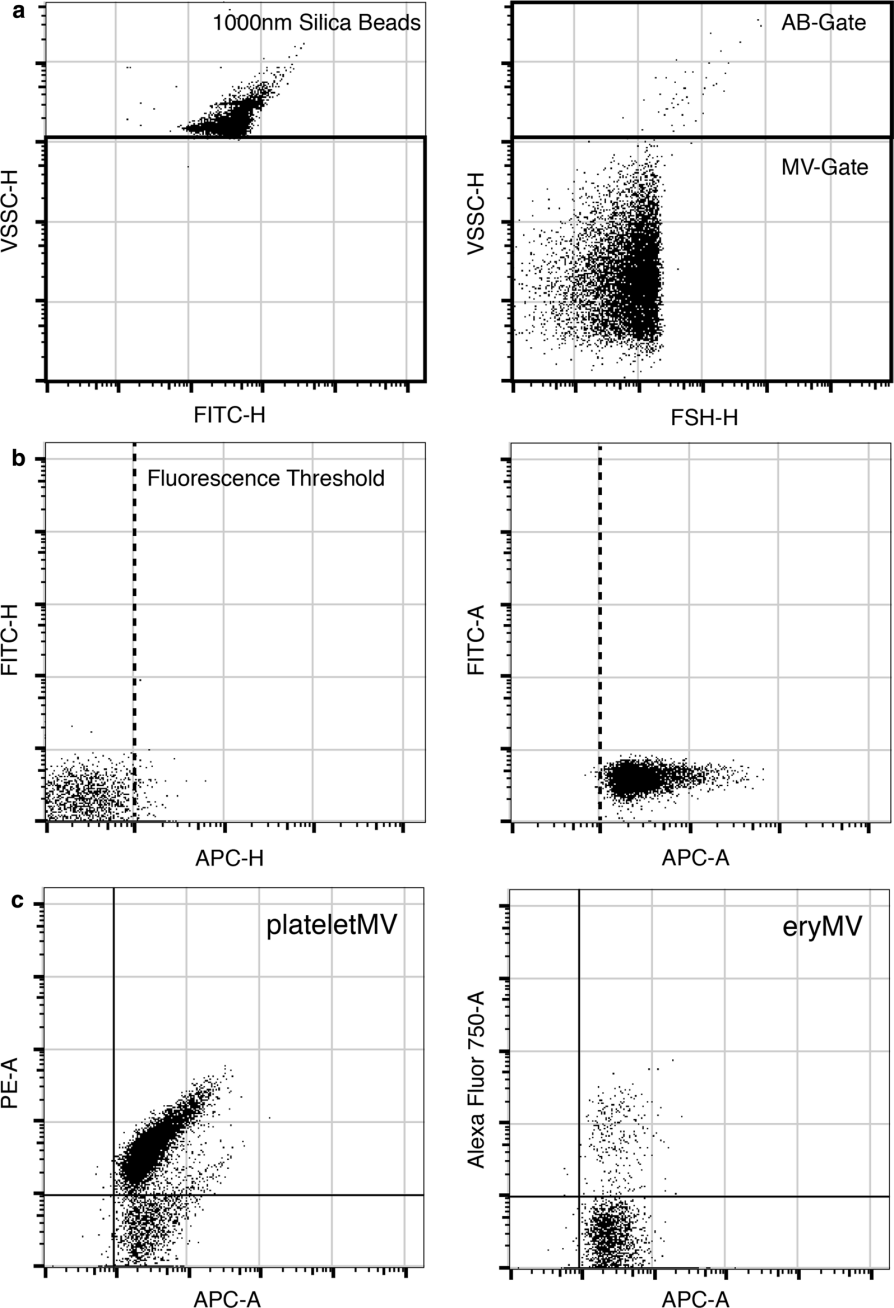
Flow cytometric setup and gating strategy. Green fluorescent silica beads (1000 nm) were used to define the side scatter properties and the microvesicle (MV) gate was set right underneath the silica bead population (left panel). Using an unlabeled plasma sample, the scatter and gate settings were validated as seen in the right panel without any trigger. All events above the MV-gate were assumed to be larger than 1000 nm and were separately analyzed as apoptotic bodies (ABs). **a** Next, the fluorescence trigger was set for the APC-channel (Annexin V (AnnV)) using an AnnV-stained plasma sample without calcium, preventing calcium-dependent labelling of AnnV (left Panel). The fluorescence trigger was set and validated using an AnnV-labelled plasma sample (right panel; **b**). The gating strategy of CD41 + MVs (platelet-derived MVs; left panel) and CD235 + (erythrocyte-derived MVs; right panel) (**c**)

For immunolabeling, samples were thawed in a waterbath at 37 °C and were immediately processed as described elsewhere [15]. Different panels were used to identify specific MV subsets: PE-labeled anti-CD41 (platelet-derived MVs/ABs), APC-AlexaFluor750-labeled anti-CD235a (erythocyte-derived MVs/ABs), PE-labeled anti-CD15 (myeloid-derived MVs/ABs), and PE-labeled anti-CD31/CD54/CD146 (activation-dependent MVs/ABs derived from activated thrombocytes and endothelial cells). All antibodies were purchased from Beckman Coulter Gmbh (Krefeld, Germany). For sample preparation, 10 µL plasma was diluted in 50 µL PBS and stained with the respective antibodies for 2 h in the dark at room temperature. In a second step, Cy5-labeled annexin V (AnnV; Biovision, Milpitas, CA, USA) was added to the sample and diluted with calcium binding buffer, resulting in a total volume of 500 µL, and incubated for 1 h in the dark at room temperature. 2 ATU/mL of recombinant hirudin (Sigma Aldrich, St. Louis, MO, USA) was added to the calcium binding buffer to prevent clot formation. Diluted binding buffer was sterile filtered through a 0.2 µm mesh to reduce background noise. Prior to staining, the antibody mixture was centrifuged at 20,000×*g* for 30 min to remove fluorescent particles as described elsewhere [16]. Flow cytometry was performed using a CytoFLEX S flow cytometer (Beckman Coulter Gmbh). For calibration of the violet side scatter (405 nm), green fluorescent silica beads (1000 nm, Kisker Biotech, Steinfurt, Germany) were additionally used to define an MV/AB gate based on the scatter properties. MVs were defined to be smaller, ABs to be larger than 1000 nm. The setup and gating strategies are shown in Fig. 1. Enumeration of vesicles was performed using volumetric measurement of the CytoFLEX S (events/µL). All persons conducting the flow cytometric characterization were blinded to the study groups.

### Tissue factor activity

For the vesicle-associated TF activity measurement, vesicles were isolated from platelet-free plasma by centrifugation at 18,000 g for 20 min at 4 °C, washed twice with Hank’s balanced salt solution and resuspended in 200 µL of HBSA. Samples were incubated with either mouse anti-human TF antibody or a control antibody for 15 min at room temperature, and then 50 µL aliquots were added to duplicate wells of a 96-well plate. Next, 50 µL of HBSA containing 10 nm activated factor VII (FVIIa), 300 nm factor X (FX) and 10 µL CaCl_2_ was added to each sample and the mixture was incubated for 2 h at 37 °C. Activated FX (FXa) generation was stopped by the addition of 25 µL of 25 nm EDTA HBSA buffer. 25 µL of the chromogenic substrate was then added and incubated at 37 °C for 15 min. Finally, absorbance was measured at 405 nm. The TF-dependent FXa generation was determined by subtracting the amount of FXa generated in the presence of TF antibody from the amount of FXa generated in the presence of the control antibody.

### Electron microscopy

The adhesion of blood cells to the adsorbent beads was studied using scanning electron microscopy. After whole blood treatment, the adsorbent cartridges were thoroughly rinsed with isotonic saline, and the adsorbent beads were removed from the cartridges and fixed in saline solution containing 2.5 vol% glutaraldehyde (Carl Roth GmbH, Karlsruhe, Germany). Samples were dehydrated using an ethanol gradient, dried for 12 h at room temperature, sputter-coated with gold (Q150R ES, QUORUM), and analyzed with a TM-1000 Scanning Electron Microscope (Hitachi Ltd., Tokyo, Japan).

### Statistical analysis

Continuous data are summarized as mean and standard deviation. Categorical data are summarized as frequencies and percentages. Normal distribution was assessed using Shapiro–Wilk test. Normal-distributed data were analyzed using the Student’s *t* test or the ANOVA post hoc Tukey test. Non-normal distributed data were analyzed using the Wilcoxon rank-sum test or Dunn’s non-parametric comparison for post hoc Kruskal–Wallis test. Correlation analysis was performed using the Pearson correlation coefficient. Statistical significance was assumed as *p* < 0.05. Statistical analysis was performed using R 3.5.1 (https://www.r-project.org).

## Results

### Patient characteristics

A total of 18 patients (22% female) were included in the study: 9 in the intervention group and 9 in the control group. The mean age was 70.2 ± 9.9 years. In 5 cases (28%) a coronary artery bypass graft, in 4 cases (22%) a combined procedure and, in 9 cases (50%) a valve procedure was performed. We did not observe significant differences in demographic or clinical data in our study cohort. The patient characteristics are summarized in Table 1.

### Hemadsorption does not affect circulating vesicle counts or tissue factor activity

**Fig. 2:**
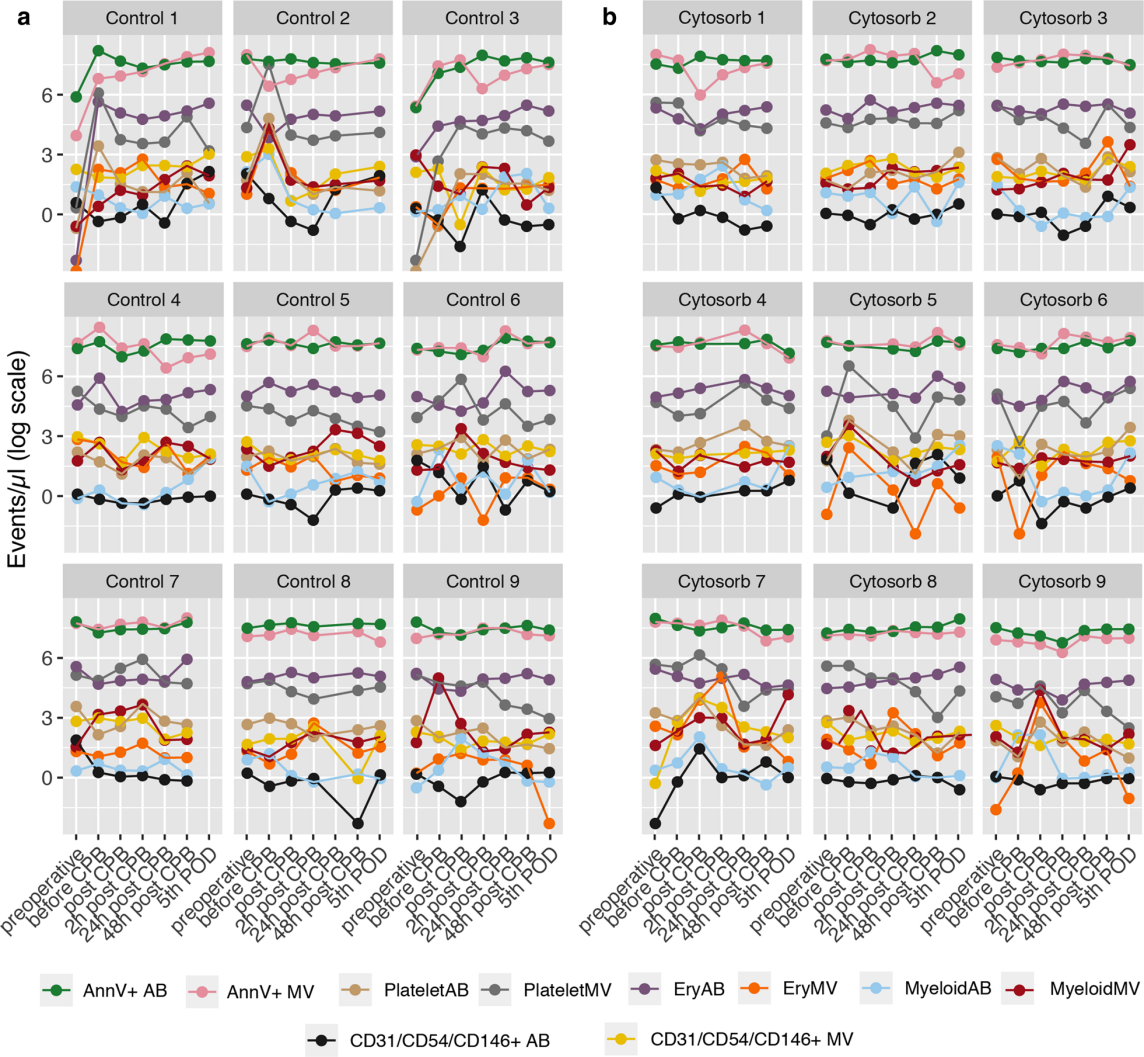
Individual count of circulating blood vesicles. Individual microvesicle (MV) and apoptotic body (AB) counts (events/µL) in plasma samples from each patients undergoing major cardiac surgery with cardiopulmonary bypass (CPB) in Control group (n = 9; **a**) and Cytosorb group (n = 9; **b**) at different timepoints

**Fig. 3:**
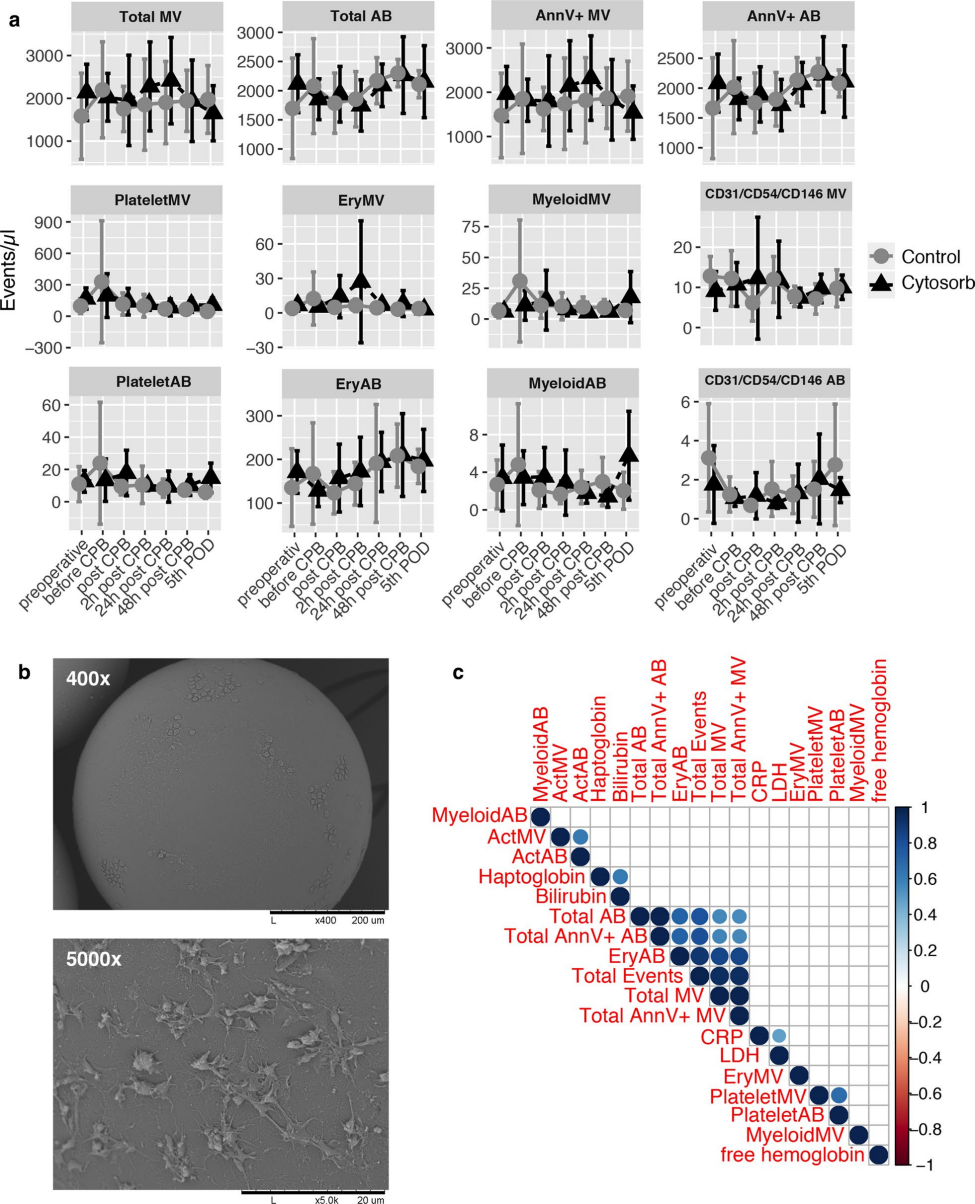
Count and hemolysis parameter correlation of circulating blood vesicles. Vesicle counts (events/µL) in plasma samples from patients undergoing major cardiac surgery with cardiopulmonary bypass (CPB) with (n = 9; CytoSorb; black triangles) or without (n = 9; control; gray circles) hemoadsorption. Samples were analyzed using flow cytometry. Data are presented as mean ± standard deviation (**a**). Scanning electron microscopy was performed to analyze a hemoadsorption column directly after use. Respective pictures are shown in ×400 and ×5000 magnification (**b**). Correlation analysis was performed to assess the link between vesicle counts, hemolysis and infection parameters. The color represents the respective rho-value and the presence of circles indicate a significant p-value < 0.05 (**c**)

First, we assessed the MV and AB count in both study populations utilizing flow cytometry. The cut-off between the two vesicle populations is based upon a physical determined scatter-property using fluorescence-marked 1000 nm silica beads. Based on this marker, we distinguished MVs (< 1000 nm) and AB (> 1000 nm) and the corresponding subset using fluorescence labelled antibodies. We found high interindividual differences in the exaggeration of MVs and ABs. Overall, we found no statistical significance in both study populations based on total vesicle count, total MV/AB count or phosphatidylserine bearing (AnnV+) MVs/ABs. Analyzing vesicle subsets, we found no significant difference in platelet-derived MV/AB, erythocyte-derived MV/AB, myeloid-derived MV/AB or CD31/CD54/CD146 MV/AB (Fig. 2). All subsets showed similar kinetics after surgery independent of the use of HA (Fig. 3a). However, based on these results, we analyzed one CytoSorb column using scanning electron microscopy. Although we observed adhesion of blood cells on the polymer beads of the CytoSorb column, this phenomenon was not reflected in the circulating vesicle count (Fig. 3b). Additionally, the TF-activity assay showed no detectable levels of TF in our plasma samples (data not shown).

### Vesicle counts correlate with High-Motility Group Box-1 levels

**Fig. 4:**
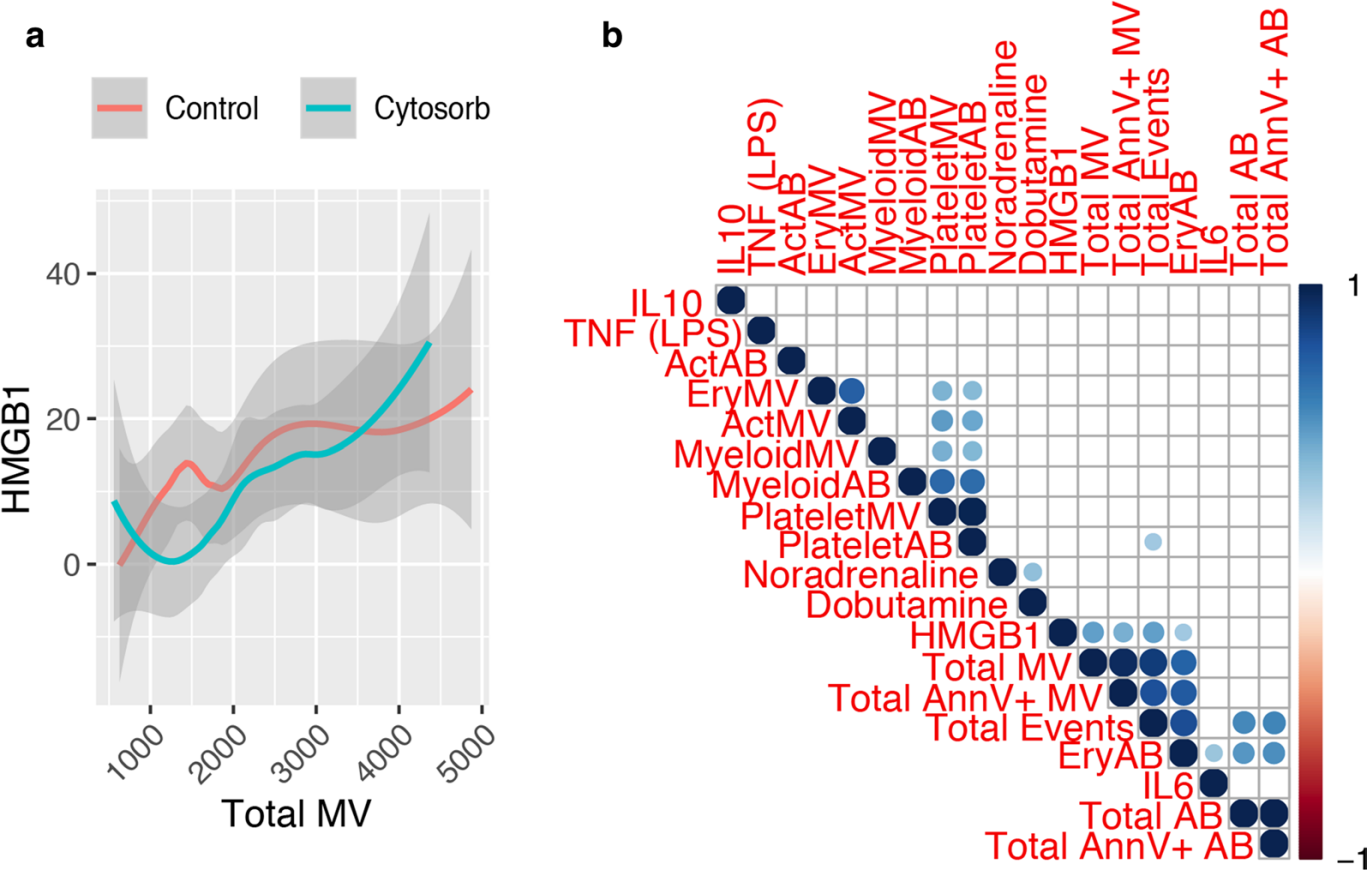
Correlation analysis of blood cytokine/alarmin levels and microvesicle count. Correlation analysis of total microvesicle (total MV) counts and HMGB1 plasma levels (**a**). Correlation plot of investigated cytokines, alarmins and inotropic drugs with MV counts. The color represents the respective rho-value and the presence of circles indicate a significant p-value < 0.05 (**b**)

Next, we aimed to assess the utility of circulating microvesicles and apoptotic bodies as indicators of inflammation and hemolysis. In our previous studies [4, 5], we found no effect on inflammation and hemolysis between patients treated with hemoadsorption compared to the control group, although a greater decrease in haptoglobin levels on postoperative day 1 was observed. Using correlation analysis, we did not detect significant correlations between MV subpopulations, haptoglobin, free hemoglobin, bilirubin, lactate dehydrogenase and C-reactive protein (Fig. 3c). However, we found that High-Motility Group Box-1 (HMGB1) is significantly correlating with the total MV (Ann + and Ann-) events (Fig. 4a) as well as with erythrocyte-derived apoptotic bodies in both groups, but not IL-6, IL-10, TNFα (after LPS stimulation). Additionally, use of inotropic drugs such as noradrenaline and dobutamine showed no correlation with MV counts (Fig. 4b).

## Discussion

Microvesicle counts during CPB using the CytoSorb HA cartridge have not been assessed before. In this study, we did not find any differences in perioperative plasma vesicle counts between patients treated with or without the CytoSorb device. Additionally, we found no significant differences in the time course of circulating plasma vesicles within each group.

Cardiac surgery, especially those utilizing CPB, is associated with the development of intravascular hemolysis, which appears to be linked with adverse clinical outcome [17]. High mechanical stress, different blood flow and pressure conditions, surface activation and cardiotomy suctioning can lead to excessive hemolysis during the perioperative course [18]. In our previous study [5], no effect on hemolysis between patients treated with hemoadsorption compared to the control group was found. However, a greater decrease in haptoglobin levels and an increase in lactate dehydrogenase on POD 1 in the control group was observed, which may be an indicator for free hemoglobin adsorption in the intervention group.

Therefore, we investigated the microvesicle as well as apoptotic body count in plasma samples of these patients and correlated our findings with established laboratory hemolysis markers. Interestingly, we found no correlation between hemolytic serum markers and vesicle counts in our patients. Hemolysis is caused by active destruction of erythrocytes, leading to the excessive release of hemoglobin as well as haptoglobin in the peripheral blood stream [18]. Thus, it is tempting to speculate that the count of microvesicles and apoptotic bodies concomitantly rises as well. In our study, we found no significant difference in platelet- and erythrocyte-derived microvesicles/apoptotic bodies in both study groups. It appears that neither the supposedly higher cellular shear stress nor the proposed “adsorbing” features have an impact on microvesicles and apoptotic bodies, at least in our study. Poon et al. observed higher platelet- and erythrocyte-derived exosomes/vesicles after initiation of CPB up to 24 h post-surgery. This might be due to the different vesicle isolation procedure, as Poon et al. used an exosome enrichment kit prior analysis [19]. Using a direct phenotyping protocol without initial vesicle enrichment, we observed no significant difference. Another study, conducted by Emanueli et al. found a significantly higher exosome count (30–90 nm) after CPB initiation compared to pre-operative samples [20]. Thus, our direct phenotyping protocol enabled us to study larger vesicles such as microvesicles and apoptotic bodies without isolation bias but might explain that we found no significant differences in the respective vesicle populations as we were unable to sufficiently detect exosomes/vesicles smaller than 200 nm via flow cytometry.

Subsequently, we were interested in the physical interactions of the CytoSorb cartridge with circulating peripheral blood cells. We investigated a used CytoSorb cartridge via scanning electron microscopy, showing cellular attachment of leukocytes on nearly all polymer beads. We additionally analyzed myeloid- and activation-derived microvesicles and apoptotic bodies but did not find differences in the vesicle counts between both groups. Furthermore, we found no difference in TF activity in both populations. This is in line with previous results, as patients undergoing CPB showed a high tissue factor dependent activity in pericardial effusion, but not in peripheral plasma [21]. In general, CPB leads to immune cell activation and release of inflammatory mediators [2]. However, these mediators do not seem to induce TF-expression on peripheral blood MVs. In our previous study, we found a long-lasting anti-inflammatory Interleukin-10 response in CytoSorb treated patients, but no statistically significant difference in proinflammatory mediators [4]. It appears that the insertion of the CytoSorb device, adding additional artificial surfaces and materials, does not boost the systemic immune cell activation in the circulation.

Lastly, we aimed to assess the utility of plasma vesicles as inflammation and hemolysis markers in patients undergoing CPB. Microvesicle counts, for example in packed red blood cells, seems to correlate with occurring hemolysis acting as quality indicator [22]. Thus, using correlation analysis, we found no significant association between inflammatory markers and hemolysis marker in our patients, nor a difference in the use of packed red blood cells and inotropic drugs. Interestingly, we found a significant correlation of HMGB1 with total MV counts in our study cohort. HMGB1 is an alarmin and is passively released upon tissue injury, necrotic cell death and cellular stress or actively secreted via immune cell activation [23, 24]. Secreted HMGB1 seems to exert immunomodulatory properties, activation inflammatory cascades via TLR4 as well as acting as immune chaperon [25–27]. Those MVs seems to play a role in immunoregulation during burn injuries [28]. However, the role of HMGB1-bearing vesicles during CPB needs to be further evaluated.

This study has several limitations. We were not able to include all patients from the main clinical study, but only a subgroup of 18 individuals. A higher patient number is needed to detect significant differences between the investigated cohorts. Additionally, with our flow cytometric method we are not able to sufficiently detect and quantify exosome levels in our study. Finally, we only investigated the count and TF-dependent procoagulatory function of circulating plasma vesicles, not the carried content inside of the vesicles (e.g. microRNA).

## Conclusions

This is the first study evaluating the impact of HA on circulating microvesicles and apoptotic bodies in patients undergoing CPB. Adding additional artificial surfaces to the CPB-circuit with the use of the HA device had no effect on the systemic immune cell activation in the circulation. However, we observed adhesion of blood cells on the polymer beads of the CytoSorb column. Larger studies are needed to assess and clarify the effect of HA on circulating vesicle counts and function.

## Data Availability

The data that support the findings of this study are available in anonymized form from the corresponding author on reasonable request and after agreement with the local ethics committee.

## Abbreviations

AB: Apoptotic body
AnnV+: Annexin V
CD: Cluster of differentiation
CPB: Cardiopulmonary bypass
EV: Extracellular vesicle
FVIIa: Activated factor VII
FX: Factor X
FXa: Activated FX
HA: Hemoadsorption
MV: Microvesicle
POD: Post-operative day
TF: Tissue factor
